# Response of vegetation to submergence along Jingjiang Reach of the Yangtze River

**DOI:** 10.1371/journal.pone.0251015

**Published:** 2021-05-07

**Authors:** Guoliang Zhu, Yitian Li, Zhaohua Sun, Shinjiro Kanae

**Affiliations:** 1 State Key Laboratory of Water Resources and Hydropower Engineering Science, Wuhan University, Wuhan, China; 2 Department of Civil and Environmental Engineering, Tokyo Institute of Technology, Tokyo, Japan; Duy Tan University, VIET NAM

## Abstract

This work explores the changes in vegetation coverage and submergence time of floodplains along the middle and lower reaches of the Yangtze River (i.e., the Jingjiang River) and the relations between them. As the Three Gorges Dam has been operating for more than 10 years, the original vegetative environment has been greatly altered in this region. The two main aspects of these changes were discovered by analyzing year-end image data from remote sensing satellites using a dimidiate pixel model, based on the normalized difference vegetation index, and by calculating water level and topographic data over a distance of 360 km from 2003–2015. Given that the channels had adjusted laterally, thus exhibiting deeper and broader geometries due to the Three Gorges Dam, 11 floodplains were classified into three groups with distinctive features. The evidence shows that, the floodplains with high elevation have formed steady vegetation areas and could hardly be affected by runoff and usually occupied by humans. The low elevation group has not met the minimal threshold of submerging time for vegetation growth, and no plants were observed so far. Based on the facts summed up from the floodplains with variable elevation, days needed to spot vegetation ranges from 70 to 120 days which happened typically near 2006 and between 2008 and 2010, respectively, and a negative correlation was detected between submergence time and vegetation coverage within a certain range. Thus, floods optimized by the Three Gorges Dam have directly influenced plant growth in the floodplains and may also affect our ability to manage certain types of large floods. Our conclusions may provide a basis for establishing flood criteria to manage the floodplain vegetation and evaluating possible increases in resistance caused by high-flow flooding when these floodplains are submerged.

## Introduction

Many large reservoirs have been constructed along major rivers across China for the purpose of optimizing the national energy structure, including the Xiaolangdi Water Control Project (2001), Danjiangkou Reservoir on the Hanjiang River (1973), and the Three Gorges Dam (TGD, 2003). Abundant research has shown that reservoirs contribute the most directly to fluvial ecosystems, such as by changing runoff and sediment contents downstream [1, 2]. Research on 4000 km of rivers in the United States has indicated that 60% of them are sediment-deficient, while only small parts of these rivers turn out to last sediment surplus [3]. The major factor that controls the transformation of rivers is the imbalance between sediment supplies and discharge.

Over the past 60 years, the Yellow River, which is the second-longest river in China, has lost 90% of its sediment load, particularly due to landscape engineering and terracing, as well as the construction of dams and reservoirs, from the 1970s to the 1990s. As these rivers have the most potential to widen, deepen, narrow, or shallow, depending upon the type and extent of construction, challenges to fluvial dynamics can persist for months or even decades [4]. Another concern is that their ecosystems are also altered to some extent by these projects.

Past assessments of the impacts from six Andean dams showed that, due to the reduction of sediment, phosphorus, and nitrogen from the Andes Mountains, the survival, phenology, and growth of floodplain vegetation, as well as fish yields, were greatly altered. In 2005, Forsberg et al. [5] found that the construction of hydroelectric dams in northern Brazil caused more significant damage to tree community structures than expected and caused them to degrade rapidly in both structure and composition. Furthermore, the riparian forests of the Elwha River, Washington, USA, have been shown to vary as a result of two dams. Shafroth et al. [6] proposed that a dramatic reduction in sediment supply was responsible for the geomorphic responses of channel and bottomlands, which eventually interfered with the patterns of riparian vegetation in river segments downstream of the dams.

From the perspective of hydrological discharge, the attenuation effect of dams tends to reduce the frequency of massive flooding downstream. The model developed by Khaddor et al. has proved that the Mechlawa dam of the Mghogha basin helped reduce a high peak discharge and the volume of a reference flood on the 23rd of October,2008, therefore, prevented flooding on the industrial zone near the downstream river [7]. Large volumes of sediment are impounded as dead storage, causing a sharp decline in downstream sediment loads. Immediately after the construction of a dam, the morphology of the channel begins to readjust, yet different parts evolve in different ways. After the TGD began operating, the flood peak was undercut, the dry season runoff was complemented, the sediment supply declined, and the riverbed suffered a new round of erosion. According to annual terrain measurements, the river channels have been scoured deeper, which has led to lower water levels under equal water discharge [8]. However, abundant evidence from gauging stations has also indicated that under high-flow conditions, water levels have failed to rise as expected, resulting in medium and low discharge [8–10]. In general, flood capacity has not risen or declined significantly, based on the geomorphic expansion of the high-flow section, and this has undoubtedly increased the risk of an uncontrollable flood occurring in the future [11, 12].

The TGD was put into operation in 2003, reducing the reservoir outflow to 16,000 m³/s out of 55,000 m³/s when a flood peak occurs. In 2010, the TGD sequentially intercepted floods exceeding 50,000 m³/s three times and cumulatively retained over 26 billion tons of runoff in storage. In 2012, the amount of water contained was 20.05 billion tons. The impoundment was typically used to compensate for dry seasons (i.e., November to May of the following year), allowing low-flow channels to respond. Correspondingly, flood sections are presumed to remain under low bed-shaping effects. Floodplains are exposed to air more frequently, causing both human-induced and natural occupancy, which raises the risk of uncontrollable flood damage.

Affected by the closure of a large dam on the Sauce Grande River in Argentina, the fundamental discharge and water level were restricted; thus, channel capacity was reduced owing to the encroachment of vegetation on the channel banks [13]. Additionally, El Niño events cause anomalously low precipitation in this catchment, resulting in significantly lower Amazon River discharge and an extension of the plant growth period [14]. Similarly, such an altered hydrological regime may also result in physical barriers to the dispersion of buoyant seeds distributed by water, fish, and other organisms. The near-permanent aquatic conditions at low topographies downstream of this dam have hampered the reestablishment of the surrounding igapó forest and resulted in large-scale habitat loss [15]. Given that erosion primarily occurs in the dry sections of river channels, decreases in water levels caused by sediment migration have out-distanced the elevation by riverbed armoring [16–18]. A continuously expanded wetted perimeter followed the increase in discharge, the proportion of the water level decline caused by scouring decreased gradually, and the increase in water level caused by enhanced floodplain resistance gradually became prominent.

When an unforeseen flood caused by extreme precipitation or even a major, predictable flood occurs, the flood control capacity of the river channel may be affected, and the risk of accidents increases [18–20]. Therefore, it is necessary to analyze the occupancy of the top of the main tidal flats after a reservoir begins operating, compare the development of vegetation layers at each stage, and evaluate the possible impacts of flooding. The environment in which plants grow is affected by numerous factors, including temperature, humidity, and soil moisture [21, 22]. According to previous studies, one of the most direct factors affecting the operational changes imposed by a reservoir is the change in the frequency of submergence, through which river hydrology helps control seedling germination and establishment [23]. Fluctuations in water levels on the order of 16 m in the Amazon River and its tributaries have triggered interannual flooding in the floodplains, which has driven phenological, morphological, and physiological responses by different tree species, such as anaerobic root activity and defoliation. Plant regeneration strategies have thus been affected, responding to seasonal and predictable changes in habitats. Trees in the Amazon River Basin seem to have adapted their flowering times and outcomes to maximize individual competitiveness and make use of flood cycles for pollination and seeding. Previous studies have clearly shown the performance of local flora and that the Jingjiang River has already witnessed adaptation to plant life strategies, yet few relevant studies or field investigations have been completed. The existent studies concerning vegetation growth along the Yangtze River mainly concentrate on the water-level fluctuation zone of the Three Gorges Reservoir and the Dongting and Poyang Lakes’ vegetation zones and are conducted mainly by field surveys. You et al. found that as the TGD keeps regulating the water-level of the reservoir, the vegetation of the water-level-fluctuation zone responded strategically by altering adaptive species according to height and showed great diversity with this range [24]. Another study shows that the annuals are taking the place of perennials gradually, and shrub has lost the dominant role in the inundated zone and riparian zone of the TGD [25]. Species diversity was proved to rise with elevation in the riparian zone after damming. After the TGD was put into operation, the total vegetation area in East Dongting Lake increased. The reed and forest become dominant comparing to grass due to the changes of submergence duration [26]. Researches on the plant communities of the Poyang Lake shows that variables of both water-table depth and soil moisture are strongly affecting the community distribution and thus resulted in a significant hydrological gradient [27]. Mangora et al. found that submergence time as one of the main factors will affect the mangrove’s germination by restraining photosynthetic rates [28]. However, limited studies concerning the vegetation on the downstream floodplains have been conducted primarily due to the frequent change of daily water level in space and time. As the transition zone linking the river and land, bottomlands usually show high diversity and biological productivity. The community development and partitioned mode are primarily decided by the moisture status of the soil, which could be measured by water depth, duration, frequency, fluctuation rate, and flood frequency [29]. The vegetation zoning pattern of bottomlands is likely to change year by year or season by season, making it difficult to predict the composition of vegetation communities distributed along with the water depth [30]. The alternation of the soil moisture content will stimulate or inhibit the germination of seeds, thereby changing the plant species of the local seed bank. Brittin and Brock pointed that the germination rate of seeds in autumn and spring is most affected by flooding and the lowest in summer, especially for those plants located along rivers that have completed a life cycle before the flood and take the form of seeds to avoid adverse conditions in the soil. Flood tolerance usually affects the germination of the seeds, while the proliferation of the community is decided by the fluctuation of the submerged frequency and the submerged depth in the later stage of growth [31].

In the long history, floodplains downstream of the Yangtze River have formed different landscapes and are occupied by variable patterns which remain unclassified. Ibeje and Ekwueme proposed a model framework, and the case study on the Anambra-Imo river basin proved its homogeneity [32]. Previous studies chiefly concentrate on sediment transportation, erosion adjustments, and the change of river sections. From the perspectives of floodplains, morphological adjustments are mainly studied during different periods since the TGD was put into operation, and intending to evaluate the influences of the TGD on the community distribution and diversities, it is necessary to clarify the pattern of vegetation occupancy on the floodplains as well as the quantitative indicators by which the inundation duration is affecting the vegetation coverage.

In this study, the submergence characteristics of each floodplain are explored, and the response patterns of vegetation coverage are concluded from the perspective of the intervention of hydrodynamic factors after the TGD. The Jingjiang River was chosen for three reasons: (i) eleven floodplains are separately distributed and make this segment the most complicated along the Yangtze River, (ii) sufficient hydrological data are available from at least seven hydrological stations, and (iii) Gholami & Baharlouii proves the effectiveness of the satellite images and GIS in monitoring and identifying specific characteristics of mangrove boundaries [33]. We applied the dimidiate pixel model based on NDVI using Landsat series satellite images to describe and analyze the change of the vegetation on the floodplains, and the Jingjiang reach is thoroughly contained in one remote sensing satellite image that is not cut or modified. We aimed to address two fundamental questions: (i) What are the interactions of runoff on floodplains? (ii) How does floodplain vegetation react to altered river hydrology? This study centers tightly within the Jingjiang River (riparian areas not included), where prior geomorphic assessments have focused on sedimentation and hydraulic mechanisms. Firstly, the measured topographic data and the waterfront lines along each year were used to calculate the changes in the flooding days, and then satellite remote sensing data were used to calculate the changes in vegetation coverage at the top of the bottomlands. Then three different patterns by which the runoff process influences each floodplain were concluded. Finally, based on statistical analysis, the impacts of the altered hydrodynamics in this reach were evaluated with two thresholds to describe the critical condition of vegetation growth. Conclusions draw by this research not only could provide referential suggestions for decision-makers to control the vegetation that has adverse effects on the unpredictable floods but also can help to consider the roughness pattern for the floodplain surfaces with gradient vegetation coverage in the mathematical models.

## Materials and methods

### Study area

**Fig 1:**
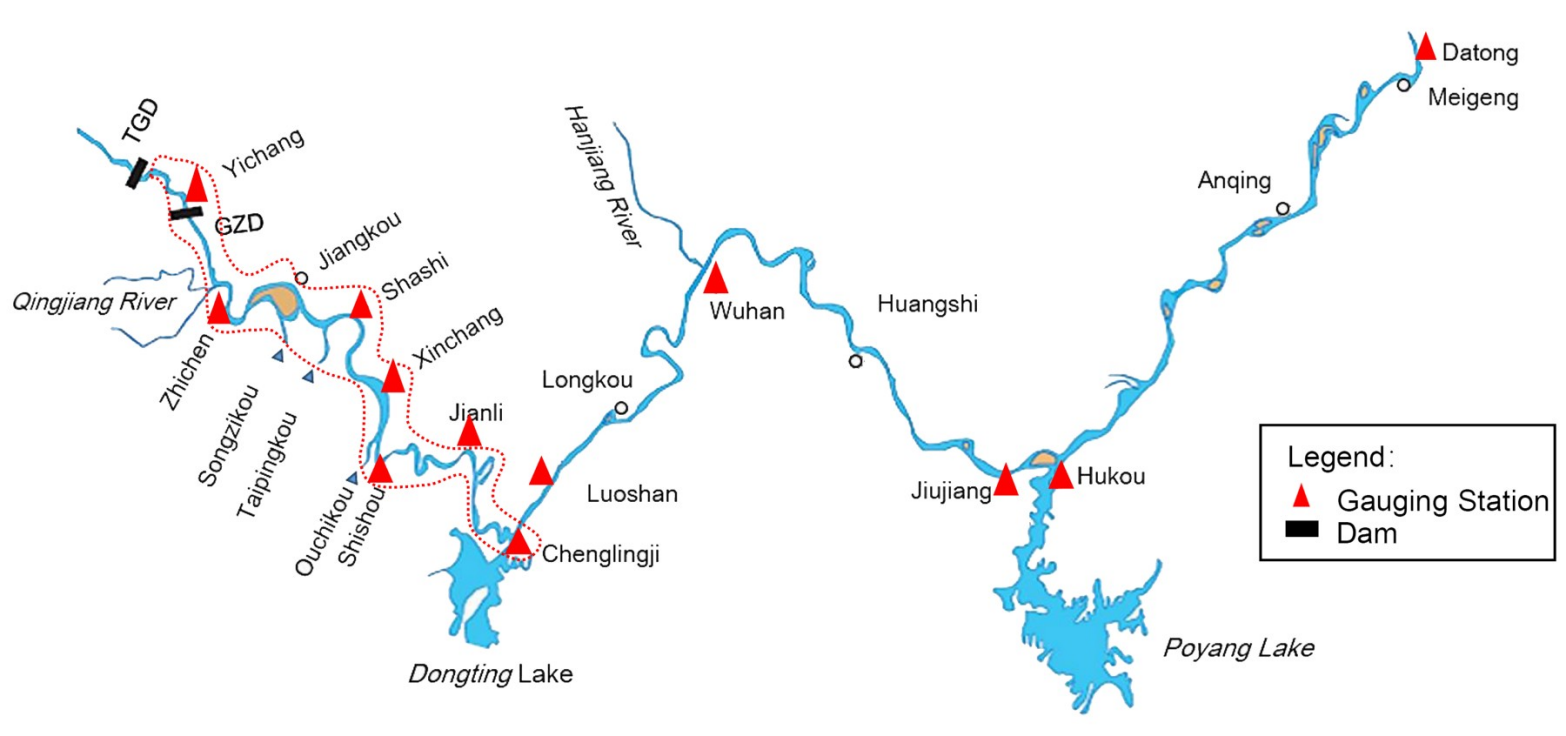
Detailed study area with gauge stations. The map was generated in ArcGIS v. 10.6 (Esri, USA).

**Fig 2:**
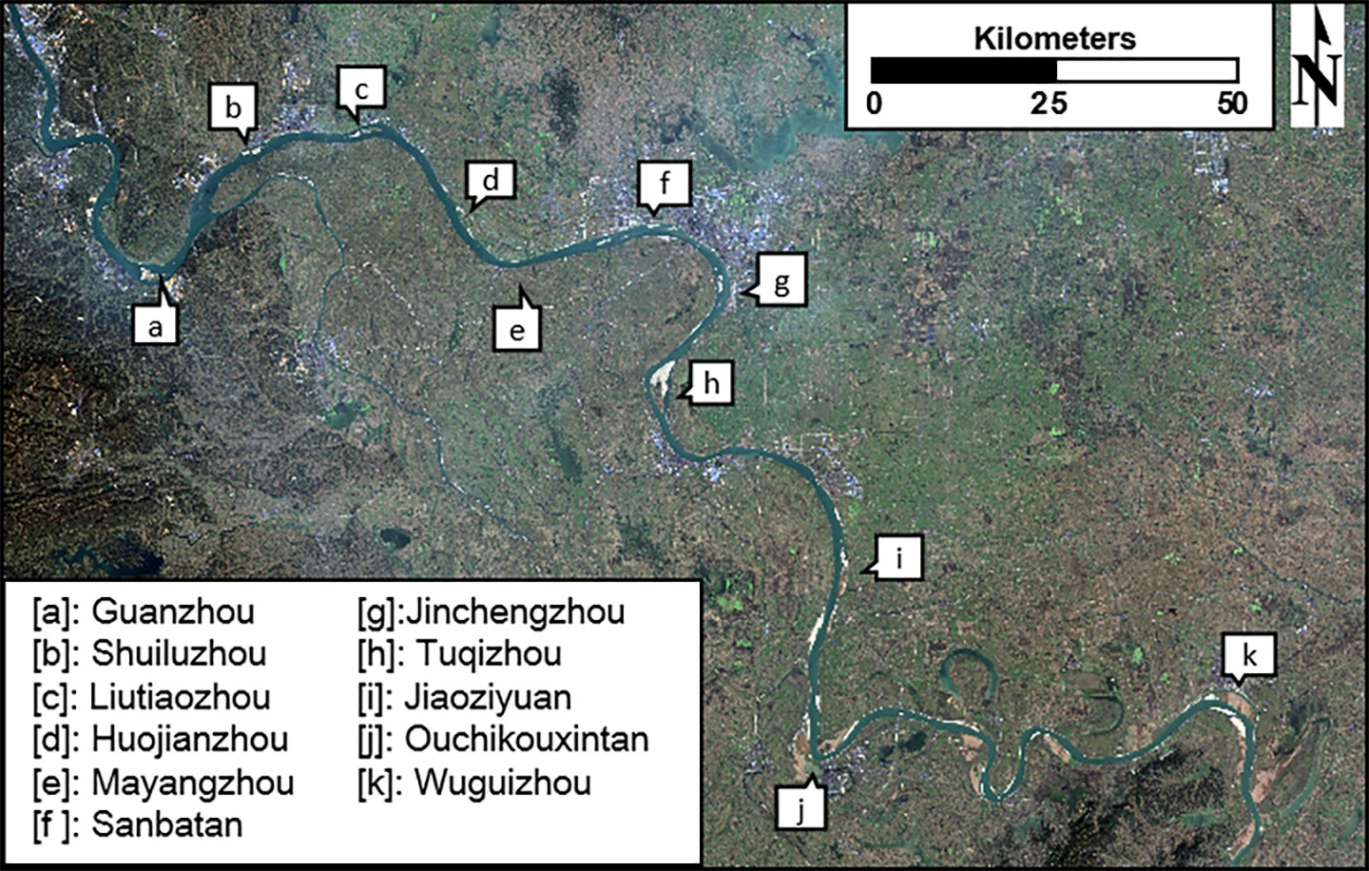
Spatial distribution of 11 floodplains along the Jingjiang Reach. **(a)** Guanzhou, **(b)** Shuiluzhou, **(c)** Liutiaozhou, **(d)** Huojianzhou, **(e)** Mayangzhou, **(f)** Sanbatan, **(g)** Jinchengzhou, **(h)** Tuqizhou, **(i)** Jiaoziyuan, **(j)** Ouchikouxintan, and **(k)** Wuguizhou. This map was calculated and generated in ENVI v.5.3 (Harris Geospatial, USA) using the OLI satellite images from Landsat-8 (paths: 124; rows: 39).

The Yangtze River Basin (Figs 1 and 2) has an area of ~1.8 million km^2^, from 24°30’–35°45’N and 90°33’–122°25’E, and is divided into upper, middle, and lower reaches by the Yichang and Hankou hydrological stations. The Yizhi Reach, which forms the transitional zone between the mountain and plain rivers, is approximately 59 km in length and has a sandy cobble riverbed. The 360-km Jingjiang Reach meanders from Zhichen to Chenlinji and is divided into upper and lower sections. Since the operation of the TGD began in 2003, the downstream channel has been scoured continuously, with the main scouring shifting from the Yizhi to the Jingjiang reach [34], and still shows an accelerating tendency due to the sandy composition of the riverbed.

The Jingjiang River (or Reach) is considered to be one of the most challenging segments of the Yangtze River due to the complexity of its tributaries, substantial bank collapse, rapid changes between river sections, and interactions with Dongting Lake. The regulation of this river directly determines both the capacities of flood control exceeding 18,000 km^2^ and shipping demands downstream. There are eleven main floodplains along the Jingjiang Reach of the Yangtze River named Guanzhou, Shuiluzhou, Liutiaozhou, Huojianzhou, and Mayangzhou, Sanbatan, Jinchengzhou, Tuqizhou, Jiaoziyuan, Ouchikouxintan, and Wuguizhou (Fig 2). The channel of the Yizhi Reach has been so fully armored that its riverbeds are mostly covered with gravel bedload, where souring is restricted. However, the Jingjiang Reach has a sandy riverbed. Thus, strong erosion occurs, and its three outfalls show no distinctive evidence of decline.

The amount of rainfall corresponds well to the temperatures across the Yangtze River Basin under the control of the monsoonal climate. Many water facilities have been constructed in recent years, including nearly 52,000 dams with a collective reservoir capacity >400 billion m^3^. More than 19,000 hydropower stations have been built and provide an installed capacity of >190 million kWh^-1^. There is no doubt that these facilities have provided great benefits in terms of flood prevention, electricity generation, and improving shipping and water supplies. However, these advantages have also changed the initial runoff processes of the affected watershed (i.e., discharge, water levels, and seasonal regimes).

The yearly amount of water from the Yichang Station in the Three Gorges Reservoir area station exhibited no obvious fluctuations from 2003–2015, when compared to the period from 1955–2002, while the evidence suggests a 1.6 billion-m^3^ mean decline every year between 1991 and 2016 [34, 35]. However, runoff primarily depends on natural climatic conditions and the flood processes within each year were evened by the presence of dams. Thus, changes mainly occurred during the storage period, from September to November, following each flood season. Previous studies have shown that discharge was reduced by 29.6% from 2008–2016 compared to 1878–1990, after each flood season and 40% in October. Meanwhile, the TGD compensates for 23 billion m^3^ of water between January and May [35].

Due to both a decline in runoff and channel reshaping, the water levels observed at gauge stations under the same flow regimes have shown a trend of decreasing after small- and moderate-sized floods and rising after larger floods. The turning point has remained near the bankfull discharge, where floods begin spreading shoreward and to the bottomlands. The volume cut and river bed erosion can both lead to the water-level fall along downstream of the TGD. In fact, only the Shashi and Xingchang stations have observed a decline of water-level exceeding 1.5 m in 2014 relative to 2003. While most of the other gauge stations show their declines within 1 m, indicating that riverbed erosion plays a more limited role in determining water levels than declining runoff [16, 17].

### Data collection

#### Hydrological data

The hydrological data used in this study included daily water levels from the gauging stations, among which the Chenlinji Station provided data on the inflow into the Yangtze River. The three outfalls were considered by using data from a hydrometrical station on the river. The hydrological and terrain data were provided by the Changjiang Water Resources Committee (CWRC) and the Changjiang Sediment Bulletins from 2003–2015.

#### Remote sensing data

Landsat 4-5TM, Landsat 7 ETM and Landsat-8 OLI satellite images from paths 124 and 39 were freely available from the United States Geological Survey (USGS) Earth Explorer website (http://earthexplorer.usgs.gov/). All image productions were systematically corrected to Level 1T for radiometric and geometric accuracy by the USGS EROS Data Center in Sioux Falls, SD. Composite color images, combining 30m red and near-infrared bands were prepared using the ENVI program (Harris Geospatial, USA). Moreover the red (R), green (G) and blue (B) bands are combined in ENVI to illustrate true color of surface condition in Fig 5.

### Analytical methods

#### Frequency of submergence

To simulate submergence times, we compared the water surface line along the reach with the terrain measured each year. The water surface line was interpolated from the water levels at the nearest gauging station. With the aim of maximizing the difference between submerging time classifications, we used natural discontinuities and divided the results into eight groups, with breaks at 30, 70, 120, 160, 210, 260, 300, and 340 days, respectively.

#### Plant occupancy

Using ENVI v.5.3 (Harris Geospatial, USA), we first employed the normalized difference vegetation index (NDVI) to distinguish between vegetation and water. A dimidiate pixel model was then used to calculate vegetation coverage. We assumed that all pixels were composed of either vegetated or non-vegetated areas, as was the resulting spectrum. Therefore, the ratios of each component should be the weights of the different cover type. We defined *S* as each pixel, *S*_*v*_ as the vegetated component, *S*_*s*_ as the soil component, and *fvc* represented the vegetation coverage used in the study.

The dimidiate pixel model based on the NDVI can be expressed as:

$$\text{S}=S_{s}+S_{v}$$ (1)

$$S_{v}=S_{veg}\cdot fvc$$ (2)

$$S_{s}=S_{soil}\cdot \left(1-fvc\right)$$ (3)

$$\text{S}=S_{veg}\cdot fvc+S_{soil}\cdot \left(1-fvc\right)$$ (4)

$$fvc=\frac{\left(S-S_{soil}\right)}{\left(S_{veg}-S_{soil}\right)}$$ (5)

Due to the linear relationship between vegetation growth and NDVI, the expression (5) can be rewrote as:

$$fvc=\frac{\left(NDVI-NDVI_{soil}\right)}{\left(NDVI_{veg}-NDVI_{soil}\right)}.$$ (6)

Where the *S*_*veg*_ represents the component of pure vegetation, *S*_*soil*_ represents the component of pure soil. *NDVI*_veg_ and *NDVI*_soil_ are the normalized difference vegetation index of pure vegetation and soil respectively [36].

**Fig 3:**
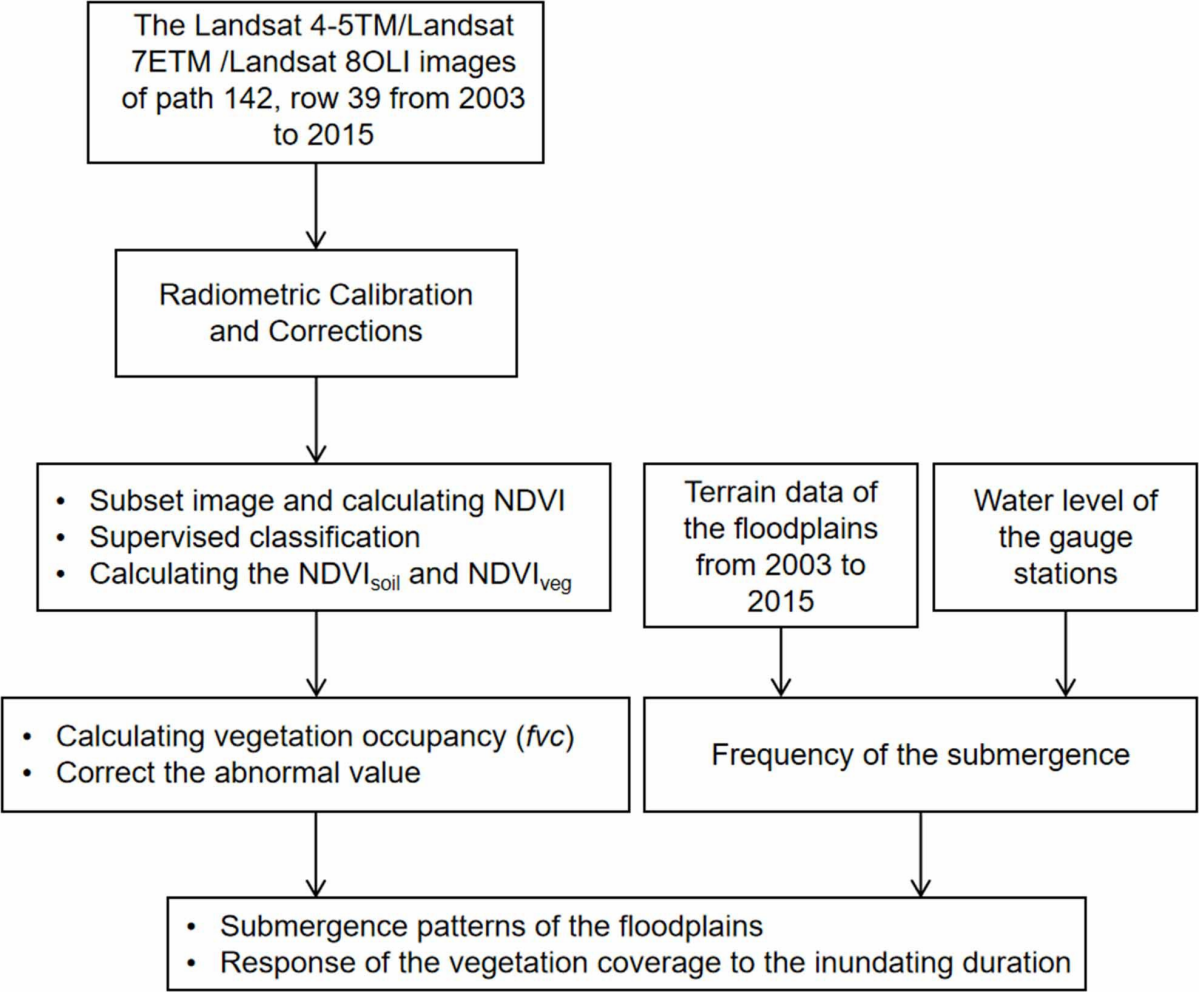
Flowchart of the research methodology.

When calculating *fvc*, the *NDVI*_*soil*_ and *NDVI*_*veg*_ determined the accuracy of the vegetation coverage. To reduce error throughout the procedure, we set pixel values summed to 2% as the minimum and 98% as the maximum and then assigned them separately to *NDVI*_*soil*_ and *NDVI*_*veg*_. Because the NDVI varies depending upon the full coverage of different types of plants, it is essential to classify the plants present in the study area. Therefore, we applied high-resolution images from Google Earth [37] and true-color remote sensing images composed of the red, green, and blue bands of the Landsat-8 OLI to sort different types of land cover. First, we interpreted and highlighted the representative plant types and then a neural network-based supervised classification scheme was used to extract additional information. Finally, outliers beyond the (0, 1) domain were eliminated before obtaining the final *fvc* results. Overall data processing methods are illustrated as Fig 3.

## Results

### Submergence frequency of main bottomlands

All of the eleven bottomlands (floodplains) were divided into three groups, labeled as Group I, Group II, and Group III, based on the primary relationships that characterized their elevations and water levels. We considered areas that were exposed during the end of every year, from mid-October to December, with the goal of maximizing the information derived from observing vegetation. When the water level exceeded the elevation of a floodplain, it was considered to be inundated by flooding (i.e., submerged).

#### Group Ⅰ —high elevation

**Fig 4:**
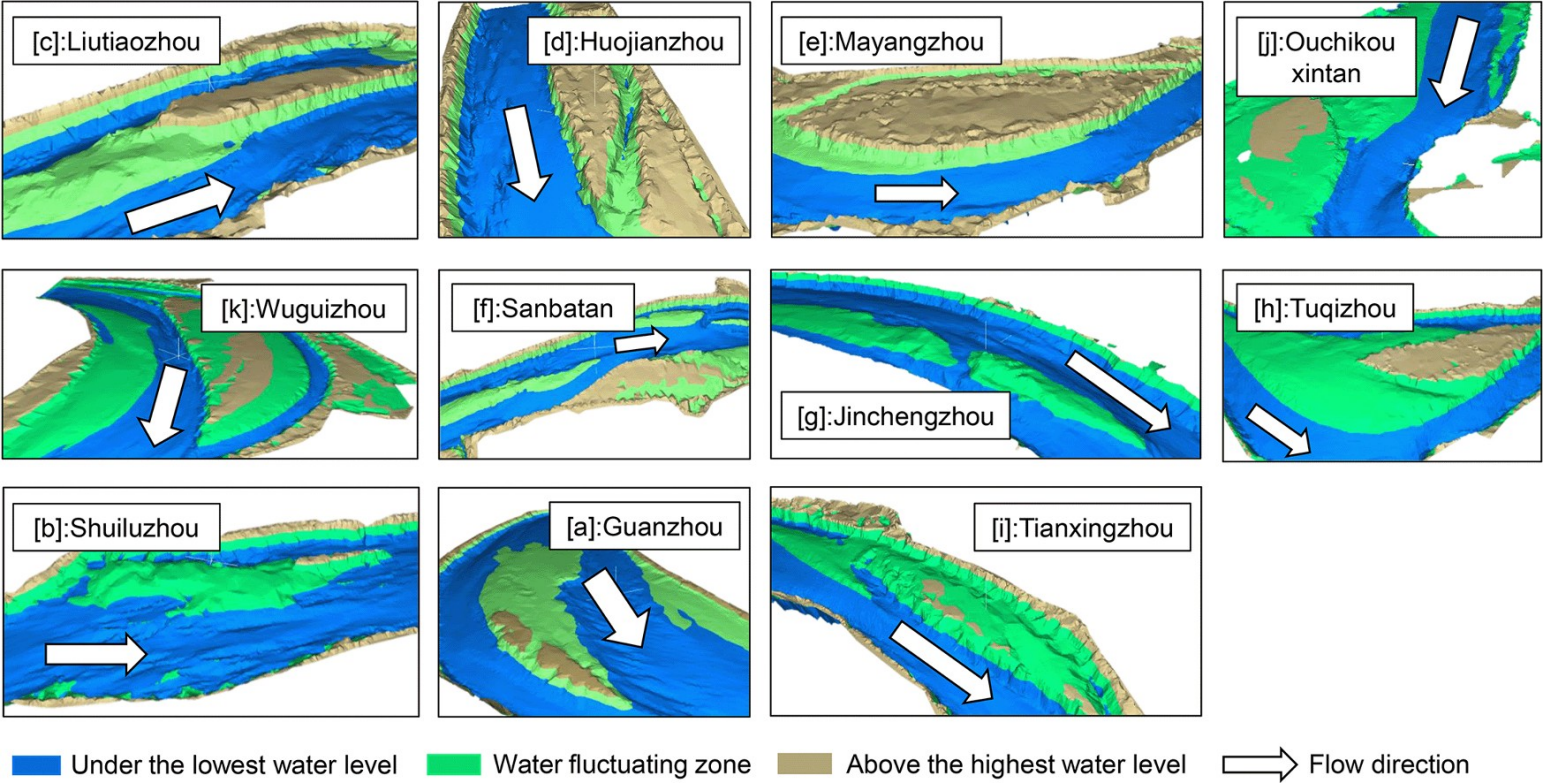
Map of the range of water-level fluctuations for each floodplain. The overall geomorphology is illustrated and was generated using Mike21 program (DHI, Denmark) based on terrain and hydrological data of 2013.

**Fig 5:**
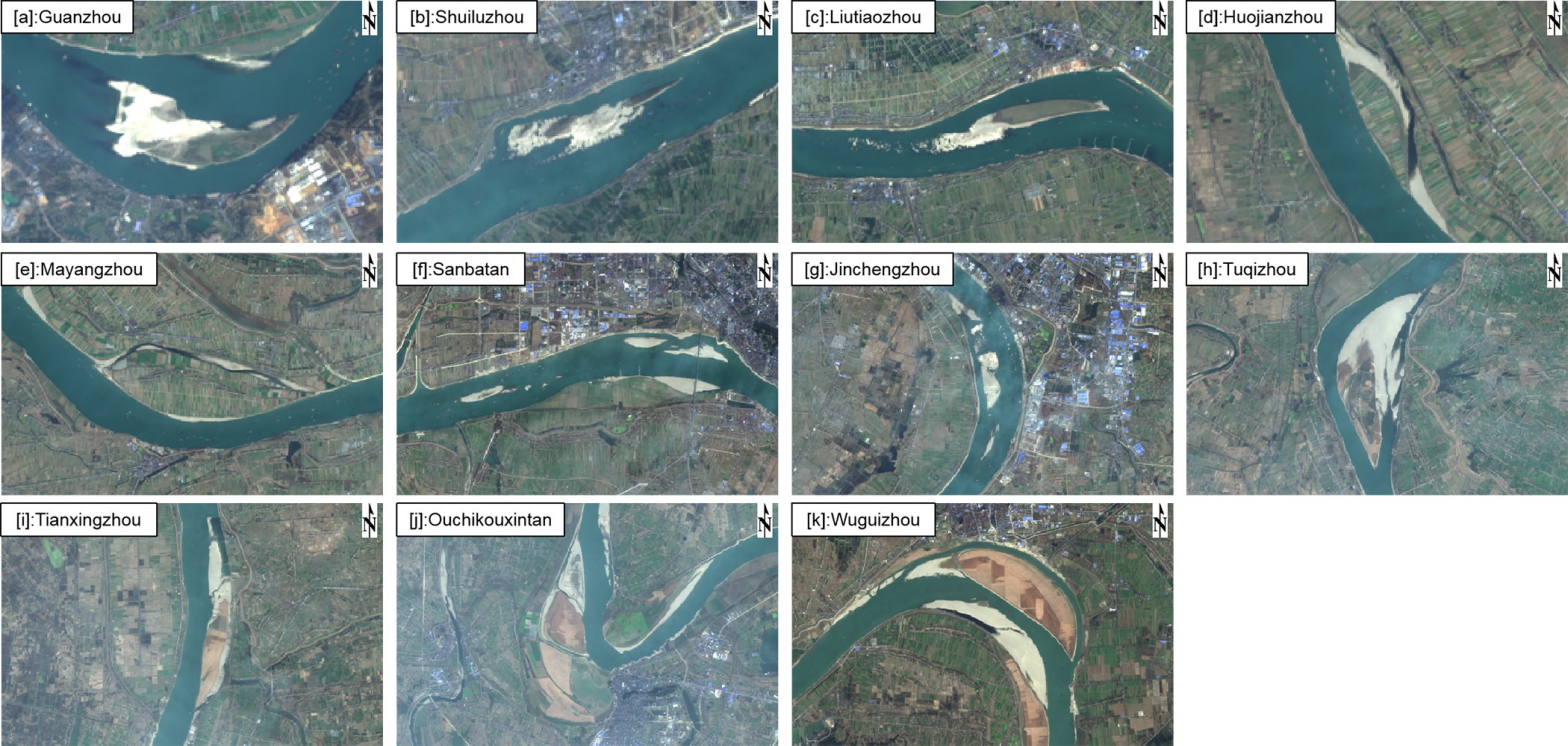
Views from Landsat 8OLI images of each floodplain on 2017/12/24 generated in ENVI 5.3 using optimized linear. These maps allowed us to distinguish specific categories of land use and to examine the accuracy of supervised classifications during the calculation of *fvc*.

The floodplains representative of Group I were Liutiaozhou, Huojianzhou, Mayangzhou, Ouchikouxintan, and Wuguizhou. This group contains bottomlands with relatively high-elevation terrain, making it difficult for floods to reach the top of the banks (Fig 4). Consequently, such lands are easily occupied for human activities, such as shelters, agriculture, forest areas, and animal husbandry (Fig 5).

**Fig 6:**
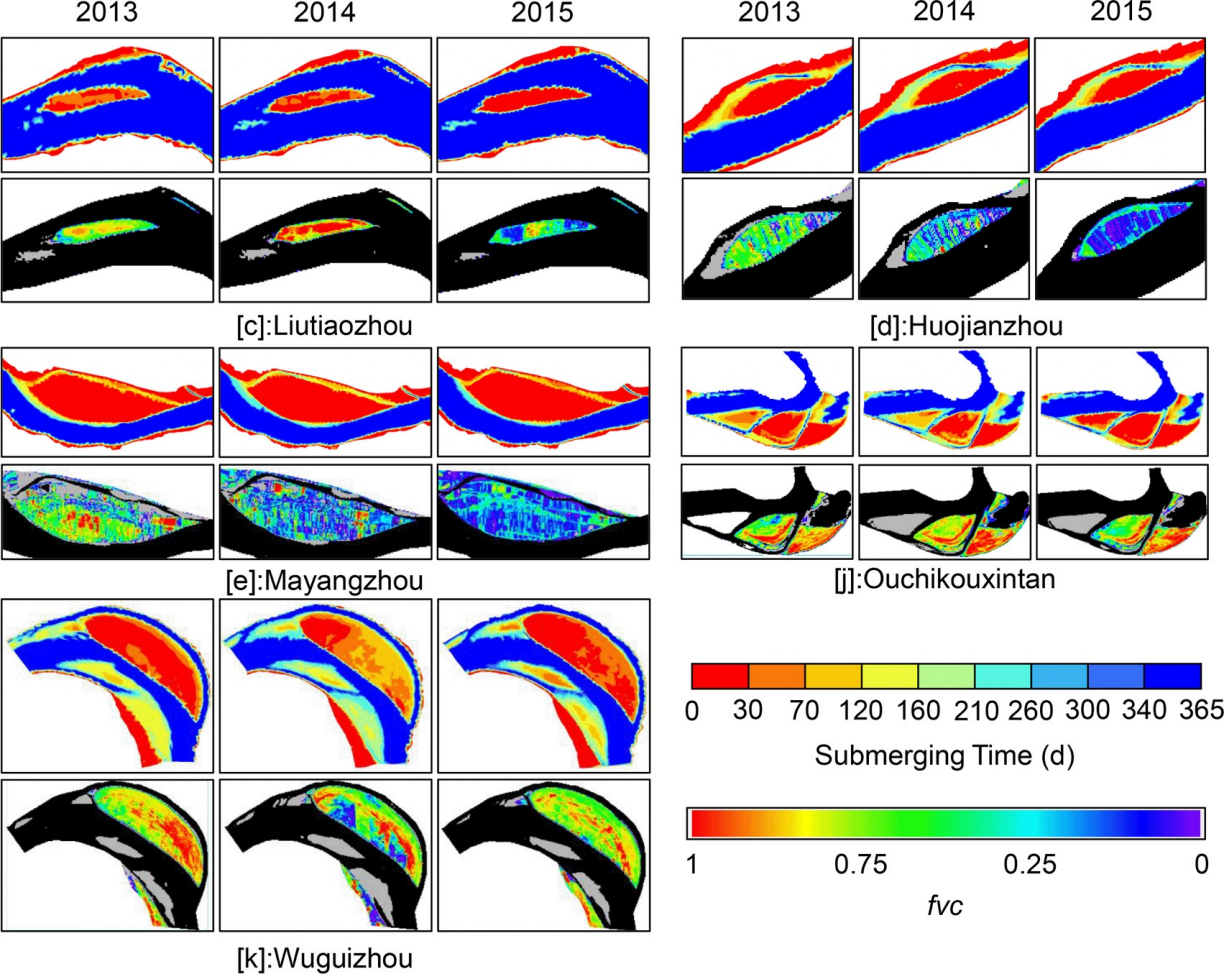
Change of submerging time (up) and vegetation coverage (below) of Group I. Black and gray parts the in *fvc* are the water surface and bare lands.

**Fig 7:**
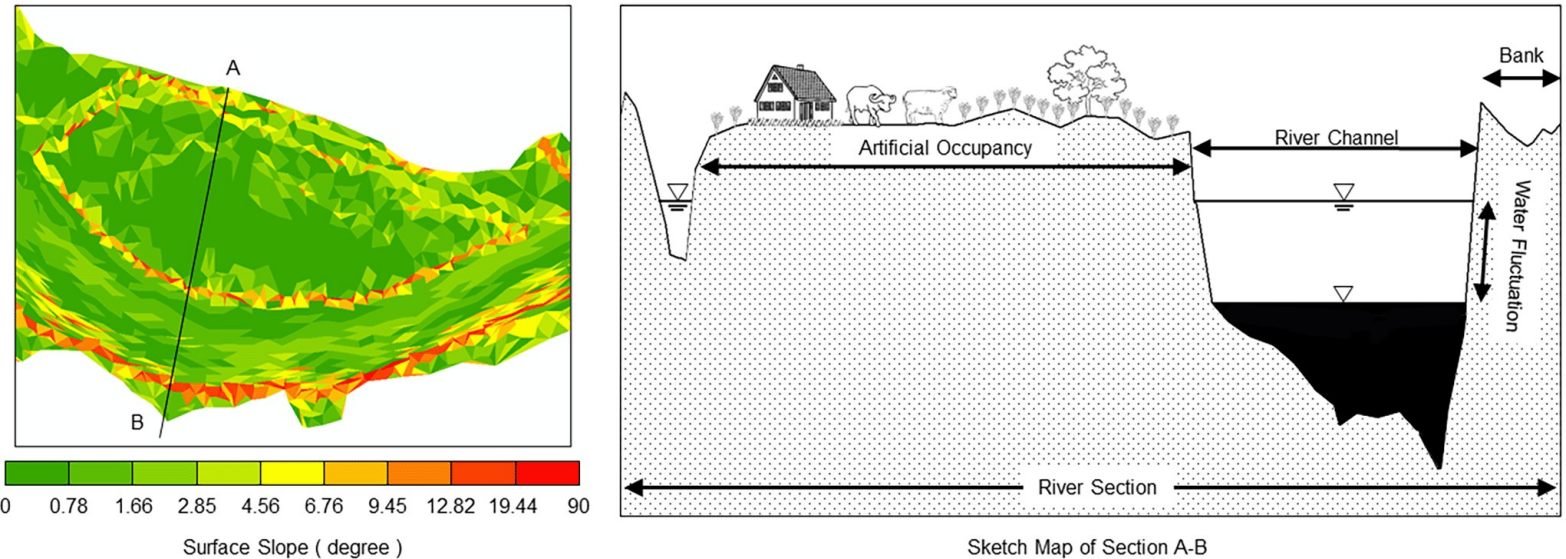
Surface slope of Mayangzhou (left) and map of the section marked A, B (right). The right graph shows the pattern of human occupancy upon the upper bottomlands and the relationships between runoff and vegetation.

Figs 4E, 6E and 7 show that the top surface of Mayangzhou is broad and flat, with a gentle slope, and water levels fluctuate within a limited range. In general, vegetation coverage is mainly determined by natural succession throughout the year rather than by runoff. Judging from Google Earth imagery (Fig 5E), many agricultural and forest lands are spread over the top of this floodplain, with a tiny proportion of residential houses. The occupancy pattern is shown in Fig 7, and the 85° edge surrounding Mayangzhou is shown to be an ideal place for agriculture.

Similar to Mayangzhou, Liutiaozhou (Fig 6C) has a lower zone where the extent of flooding varies, while the top remains isolated from runoff. Anthropic traces can be seen on the top, with spreading residential areas (Fig 5C). Due to human activities, the plant community depends mainly on certain crops. Along with other floodplains in Group Ⅰ, vegetation coverage showed an evident decline after yearly harvesting while left a distinguishable boundary that most likely represented ridges and tractor tracks. Based on the inundating days from 2003–2015, the top of the bottomlands was flooded for <30 days each year (Fig 6). No obvious evidence of uniform vegetation types was discovered, yet boundaries remained sharp and clear.

#### Group Ⅱ —low elevation

**Fig 8:**
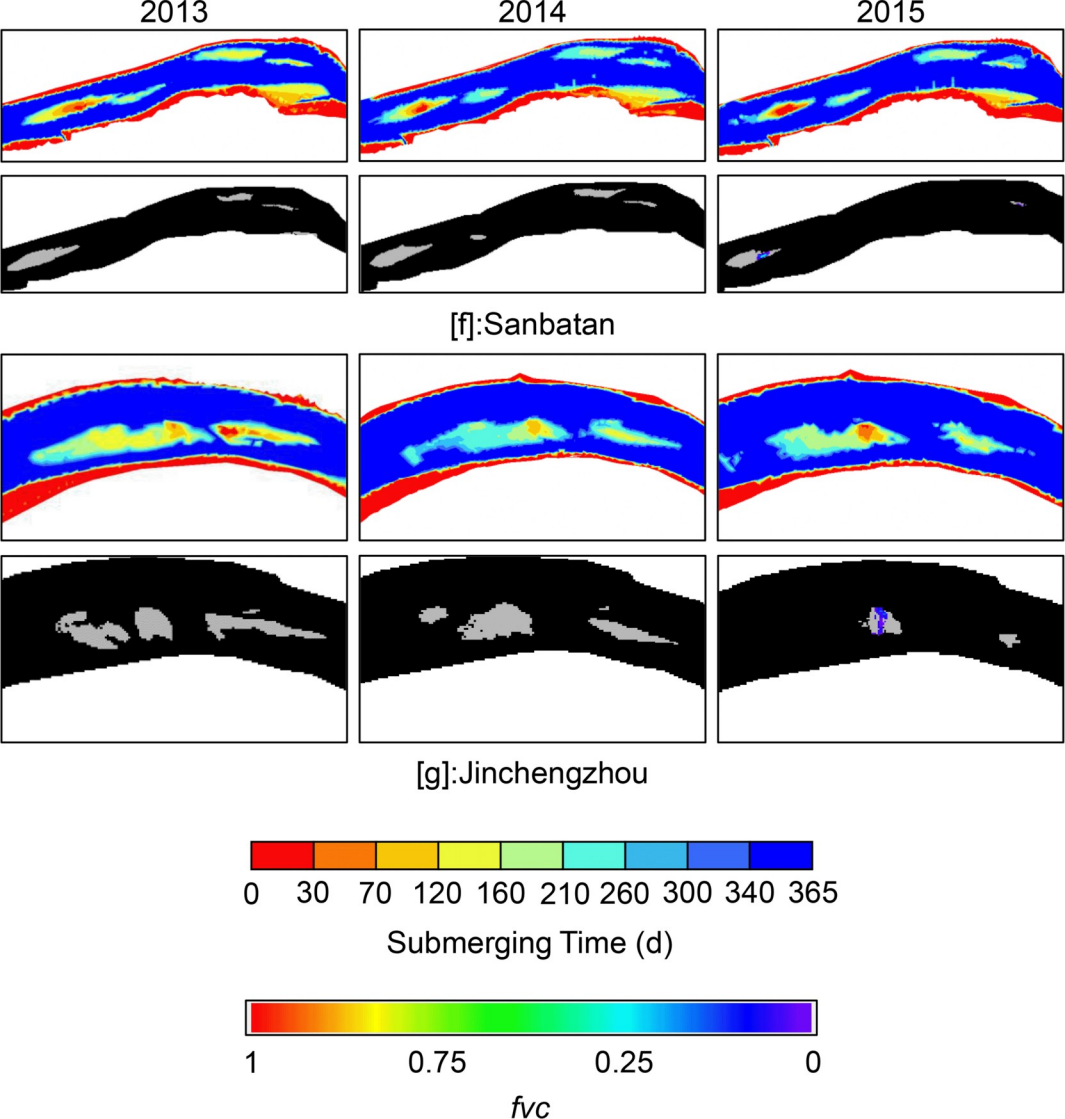
Change of submerging time (up) and vegetation coverage (below) of Group Ⅱ.

Group II included the low-elevation floodplains of Sanbatan and Jinchengzhou. Fig 8 shows that the dominant submergence time was ~160–210 days and may extend to 260 days on the edge area due to the low terrain. During the study period, apart from minor areas near the heads of Sanbatan and Jinchengzhou in 2015, there was barely any large-scale vegetation detected (Figs 5F and 5G and 8). Moreover, vegetation remained limited (*fvc*<0.25) based on calculations in 2015. Thus, the inundation time failed to fully meet the needs for plants to grow from seeds, and cover the floodplain.

#### Group Ⅲ —variable elevation

**Fig 9:**
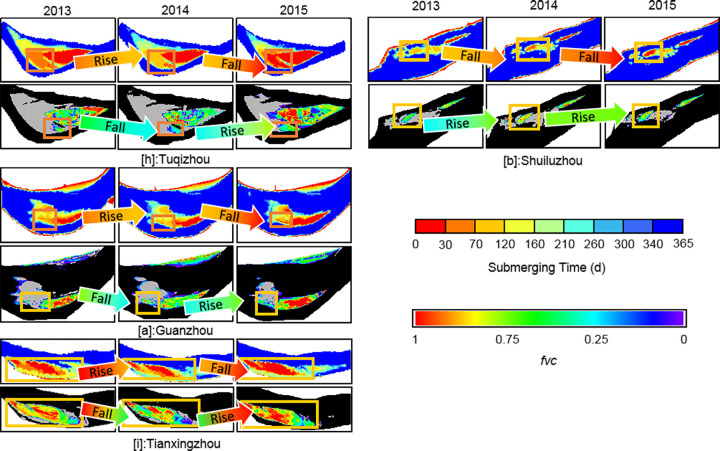
Change of submerging time (up) and vegetation coverage (below) of Group Ⅲ. The frames mark places where plants grew within the range of fluctuating water levels. Other parts were either unvegetated or above the maximum water level in each year.

Unlike the bottomlands of groups I and II, those belonging to Group Ⅲ exhibit strong correlations between plant occupancy and submergence time. Tuqizhou, Shuiluzhou, Guanzhou, and Jiaoziyuan are in this group. Despite some small places within them being similar to groups Ⅰ and Ⅱ, the majority of bottomlands in Group Ⅲ lie within the range of flood variability (Fig 4). The timeworn part of Tuqizhou (shown in red in Fig 4) is high enough for human occupancy and thus provides the proper circumstances for shelters and agriculture (Figs 4H and 5H). Meanwhile, the emergence time of newly formed upriver areas framed in Fig 9H was sufficiently spread from 260 to within 30 days. The majority of upriver areas were flooded for 30–210 days in 2013 and a total of up to 260 days closer to the upstream edges. The colors shown in Fig 4 for the same place changed from green to blue as a result of heavier flooding in 2014. However, in 2015, those inundated for 70–120 days in 2014 were flooded for just 0–30 days, similar to the older sections. Consequently, plant occupancy extended and deepened.

For Shuiluzhou (Fig 9B), the exposed areas are separate, and the older parts minimally engage with the stream (Fig 4B. However, plants have grown since 2013 in the middle portion shown in Fig 9B despite the fact that the submergence time ranges from 70–120 days. Although no vegetal expansion was observed by 2015, the plant density improved as a result of the reduced inundation frequency, from 70–120 to 0–30 days.

The older part above the water-level fluctuation zone of Guanzhou (Fig 9A) appears the same as those in Group Ⅰ. Based on the Google Earth images (Fig 5A) and *fvc*, the upriver section in the frame of Fig 9A experienced a period of new plant growth from 2013–2015 as a consequence of aerial longer. Notably, these new plants matched those areas that flooded for a maximum of 70–120 days, while low concentrations of plants were observed near the edges of Guanzhou. The remaining portion was underwater for 120–260 days, meaning that it did not meet the prerequisites needed for plant growth.

For Jiaoziyuan, in which runoff flows from the right due to a steep slope (Fig 4I), the boundary between the main channel and vegetation is clear and distinct (Fig 5I). Moreover, the vegetated region area remained ~10 km^2^ within the zone of fluctuating water levels, thus providing a convenient illustration of the *fvc* under the influence of runoff. In comparison with 2013, greater flooding in 2014 led to a more extended period of submergence and contributed to the decline of vegetation. As shown in Fig 9I, the color in the frame changed to green from red and yellow, indicating that vegetation coverage was reduced and then returned to red in 2015. Parts of some areas that emerged in less than 30 days then did so from 30–120 days, as time proceeded to the winter of 2014. In contrast, owing to the decreasing runoff in 2015, areas flooded for 0–30 days increased, as did the higher vegetation density of Jiaoziyuan.

### Changes in vegetation coverage

To summarize the response of vegetation to inundation time, the vegetal density was illustrated from 0 to 1 (purple to red in figures). It was found that almost every red zone (of vegetal density) existed where the submergence time was within 30 days (Table 1). However, the opposite did not hold true, as there were bottomlands like Huojianzhou (Fig 5D) in Group Ⅰ, which were instead occupied by human activities. Two exceptions to the overall trend included Liutiaozhou and Wuguizhou, where the highest coverage appeared in the zones experiencing 30–70 days of inundation; both belonged to Group Ⅰ (Fig 6C and 6K).

**Table 1:** Specific ranges of submergence times in which most of the highest and lowest vegetal coverage appeared.

Group	Bottomlands	Submerging time (d)	
		Max. coverage	Min. coverage
Ⅰ	Liutiaozhou (c)	30–70	0–30
Ⅰ	Huojianzhou (d)	0–30	0–30
Ⅰ	Mayangzhou (e)	0–30	0–30
Ⅰ	Ouchikouxintan (j)	0–30	0–30
Ⅰ	Wuguizhou (k)	30–70	30–70
Ⅱ	Sanbatan (f)	0–30	0–30
Ⅱ	Jinchengzhou (g)	0–30	0–30
Ⅲ	Tuqizhou (h)	0–30	70–120
Ⅲ	Jiaoziyuan (i)	0–30	70–120
Ⅲ	Guanzhou (a)	0–30	70–120
Ⅲ	Shuiluzhou (b)	0–30	70–120

**Fig 10:**
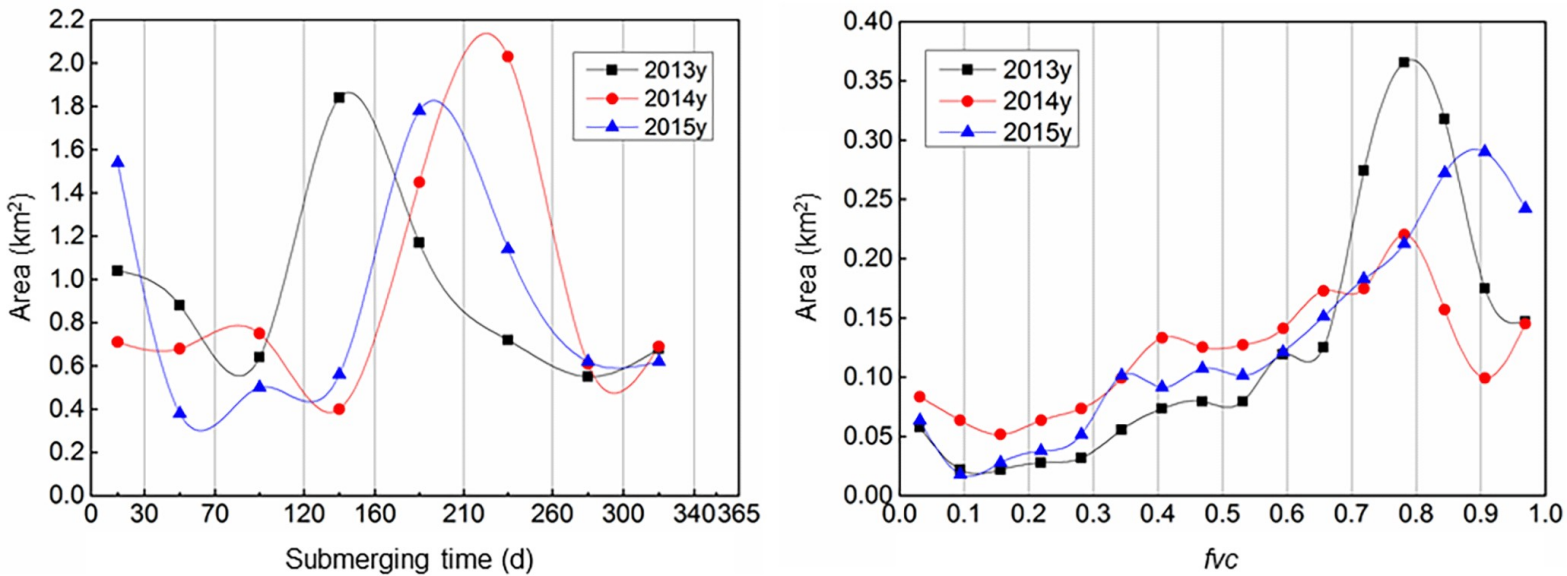
Grading curves of submergence time and *fvc* from 2013–2015 at Jiaoziyuan. Runoff in 2014 increased the likelihood inundation and thus resulted in more areas with low *fvc* values (in the range of 0–0.65); 2013 and 2015 exhibited the same patterns.

As shown in Table 1, the maximum inundation time for low-density coverage was 70–120 days under natural conditions and accorded with the pattern observed in Group Ⅲ. Meanwhile, most cases in groups I and II shared the same threshold value of 0–30 days between the maximum and minimum coverage. Hypothetically, this threshold could be predominately determined by crop types on the bottomlands of Group Ⅰ, given that they were typically exposed to the air. The curves in Fig 10 show the tendencies of both variables, where the peak coverage values show the same trends as submergence times.

**Fig 11:**
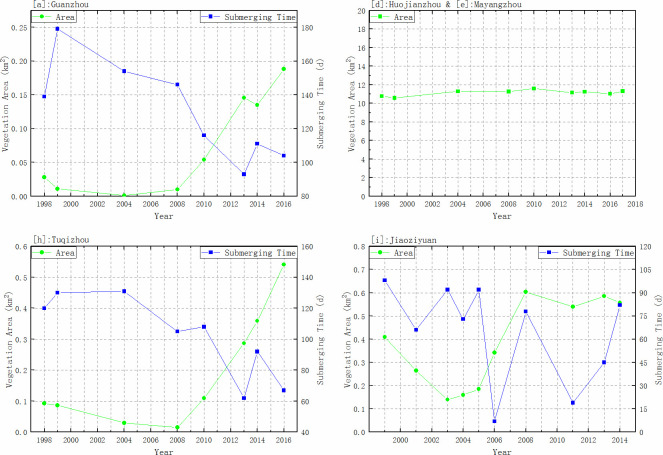
Vegetation area change of Huojianzhou and Mayangzhou, Guanzhou, Jiaoziyuan and Tuqizhou.

The result above has clarified the three patterns of submergence of all the research areas. Thus we took representative samples from each group and calculated the inundation duration and vegetation area chronologically, as shown in Fig 11. The area of Huojianzhou and Mayangzhou remain basically unchanged as it has reached the maximum of the surface and has been highly occupied by humans before the dam construction. Due to the extremely low submerging time (<5 days), the submergence curve was not plotted. For Guanzhou, Jiaoziyuan, and Tuqizhou, we choose the variable places inside the framed places in Fig 9 and found that both Guanzhou and Jiaoziyuan had the minimum vegetation area around 2004, and then the vegetation started expanding. Moreover, before 2010 the plants on Guanzhou and Tuqizhou kept low coverage and a small portion of the whole surface, and the turning point appeared within 2008 to 2010. There was a slight decline in Guanzhou in 2014, consistent with the increased submerging time from 93 days in 2013 to 111 days in 2014. Due to the relatively lower elevation of Jiaoziyuan, vegetation started the expansion earlier in 2006 when encountering the exceptionally low water discharge [38].

**Fig 12:**
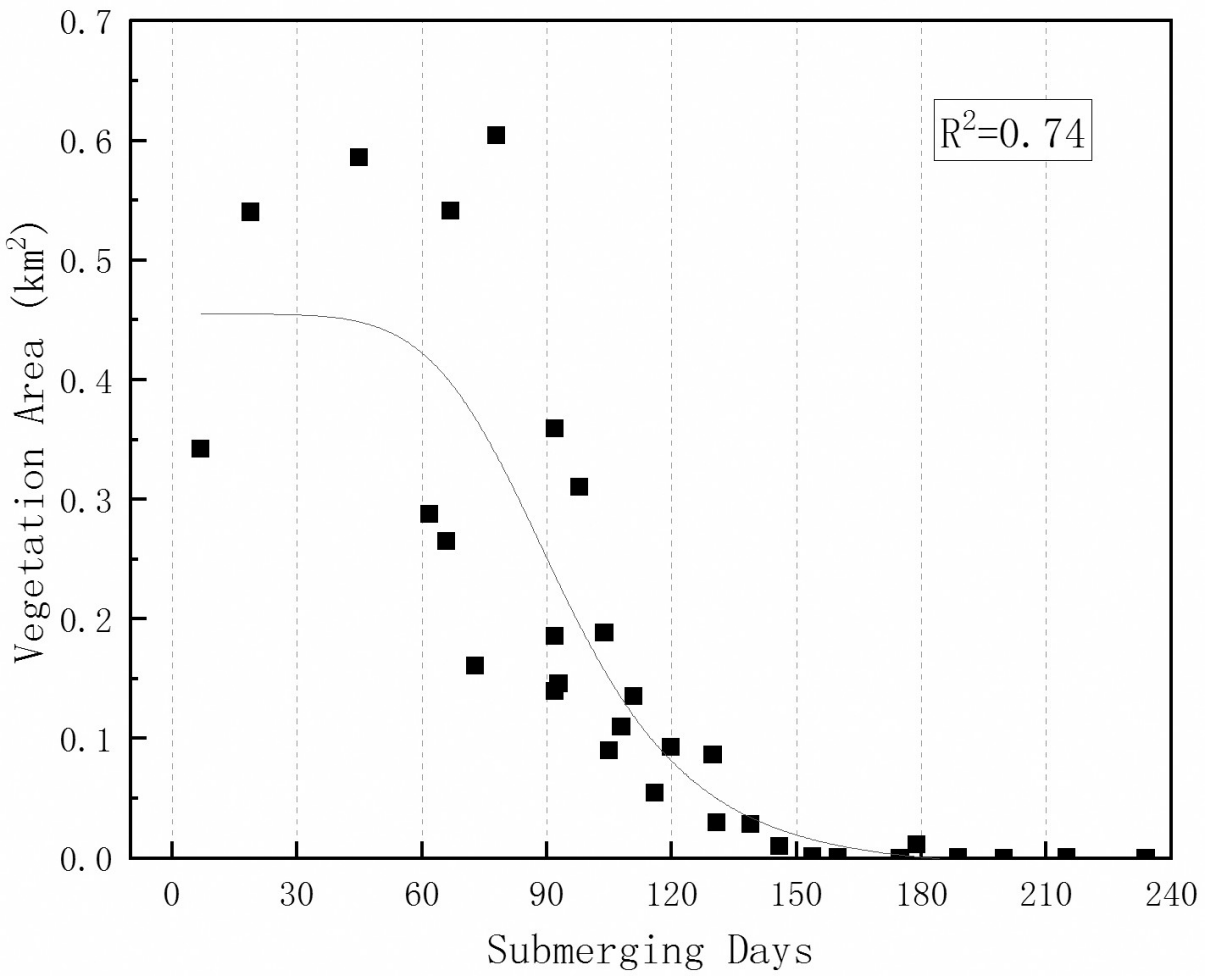
Vegetation cover area plotted as a function of flood duration in a year. The nonlinear fitting curve was calculated and R = 0.74(p<0.05).

In addition to the annual change of vegetation, the scatter graph of observed plant area corresponding to submerging time within a year was illustrated as Fig 12. These values were extracted from the water-fluctuation places on Guanzhou, Jiaoziyuan and Tuqizhou.

When submerging time is longer than 120 days, vegetation area keeps under 0.05 km^2^, and vegetation coverage is low. This indicates 120 days as a threshold for triggering the vegetation occupancy, and this is consistent with the result in Table 1. Referring to the turning points in Fig 11, the corresponding inundating time is 116 days for Guanzhou and 108 days for Tuqizhou in 2010, respectively. Meanwhile, the submerging time for Jiaoziyuan keeps under 98 days, and plants continued expanding even though submergence may fluctuate within a small range. Thus the maximal vegetation area was decided mainly by sediment erosion and river section adjustment. Also, competitions among different species are likely to disturb the vegetation coverage yet to a small extent when an unusual flood interferes with the previously-formed community. As inundating time decreases into 70–120 days, the vegetation cover enters a high rate of the increasing stage. Notably, the points in the high zone become scattered due to the upper area limit of sampling places. In other words, the vegetation will keep expanding to the maximum area of the range, which is flooded for less than 120 days.

## Discussion

This study was concentrated on the Jingjiang Reach in the middle of the Yangtze River (Figs 1 and 2). After the TGD was put into operation, the original balance of sediment erosion and deposition was destroyed [34, 39]. As regulations have been continuously adjusted, a strategy of allowing medium and small floods was enacted in 2010 [40]. As was recorded, there were only 21 days during which the maximum discharge exceeded 45,000 m^3^/s at the Yichang gauge station. Previous research has shown that discharge between 2009 ab 2014 was far from that intended.

The construction of the TGD has, to some extent, ensured the safety of lowland communities during flood seasons since 2003, yet it also remained unknown whether or not channels in the lower reaches are capable of bearing inevitable heavy flooding. Therefore, we selected 2003 to 2015 to study stream dynamics in the middle of this vital watershed [41]. Substantial research has demonstrated that water levels have risen instead of declined under the high discharges recorded at gauge stations, despite the deeper thalweg and channel readjustments via new erosion. A flood that occurred in 2011 in the Mississippi River, USA, resulted in a higher water level than in floods occurring in 1927 and 1973 owing to the presence of dense vegetation on its floodplains [42]. Similar risks might arise in China as the living environment is influenced by the TGD, and there is no doubt that the medium and small flood-induced water levels have fallen.

There are many concerns surrounding the potential risks brought by continuously deepening channels in Jingjiang. With a deeper and wider channel, the Yangtze River is supposed to carry more floodwaters. Based on the statistics available from gauge stations, declines in water levels under the medium- and small-scale flooding have been observed since the TGD was put into operation. Possible causes that have been proposed to explain this include the lack of large-scale changes in sections of high discharge, more obstructive bank geometries, or higher roughness when floods reach the bottomlands [43, 44]. Taking the floodplains that are likely to be more exposed to the air into consideration, roughness above the maximum water-level tends to increase [42], thereby contributing to higher flood risks. Past studies have contributed much to our understanding of fluvial hydraulics related to vegetation, and most have been conducted using indoor channel experiments or numerical models of small-scale rivers. Comparatively, few studies have been conducted for large natural rivers, like the Yangtze, yet most support the backwater areas [45] and are able to raise water levels during massive floods. Here, we aimed to analyze this presumption and add field observations via remote sensing data from October to December from 2003–2015. Year-end images were chosen to minimize the errors due to cloud cover and standardize vegetal growing states to the same phase before low temperatures affected their coverage. Nevertheless, we were not able to capture the entire whole growth process, such as from July to September, when plant life flourishes.

When making the specific criterion for flow and sediment regimes and river bed morphology, different levels of requirements need to be evaluated. Ćosić-Flajsig et al. Established a holistic approach to defining the environmental flow, and the case study on Sutla River Basin uses the flow characteristics to meet the spawning of the *Barbus balcanicus* [46]. The conclusions drawn in this study revealed the overall influence of current hydrological conditions and the responses of plants to different runoff processes. Policymakers and researchers may use this study as a reference to control vegetal growth in floodplains and to impose boundary conditions in numerical models, where higher roughness on floodplains must be taken into consideration. Our results indicate that areas inundated for more than 120 days were barely covered by vegetation, meaning that the duration of exposure should not have met the growing requirements of local plant life. The threshold value that appeared within 70–120 days in Guanzhou and Shuiluzhou implied that submergence for more than 120 days restrained vegetal growth, although very little (*fvc* < 0.1875) vegetation was observed in minor areas of Sanbatan or elsewhere.

Additionally, submergence times of <120 days could not ensure vegetal coverage based on the findings in Jinchengzhou, where qualified fields were small and seed transmission, as well as a growth strategy, were likely affected. Ouchikouxintan disobeyed this pattern due to armoring of the riverbed and consequently led to changes in the land surface. There are also human-modified zones in Guanzhou where plants were detected despite being inundated for more than 120 days. Thus, particular circumstances demand independent consideration.

Here, we revealed how vegetation coverage changes with submergence time and found that plants grow more densely when flooded for shorter periods (within 120 days). The threshold value lies within 70 and 120 days, which could be explained by the specific types of bottomland plants. The plant cover area, which shows strong correlations to submergence of the floodplains in Group Ⅲ, remained low before 2006 or between 2003 and 2006. The dam construction in 2003 helped reallocate the flood resource, and the following riverbed adjustment happened mostly in low terrain [18], including the Group Ⅲ which explains the low cover before 2006. As the impoundment level was increased to 175 m after 2008 [8] and the flood peak was reduced, leading the hydrodynamic conditions to meet the thresholds, the plants started increasing at a high rate. Also, on the conditions that submerging time approaches towards the threshold of 120 days, vegetation cover tends to decrease significantly. Jager et al. found 40% of the whole growing season (around 70 days) as the threshold when the diversities of both under and overstory communities start decreasing on the Upper Mississippi River [47]. Furthermore, this value has consistency with the duration range of 70–120 days that we concluded based on the Yangtze River, considering that the vegetation we observe probably has a longer growing period. Moreover, the dominant tree species like *Acer saccharinum* on the floodplains in northeast North America showed their flood-dependence in being submerged for 4.5–95 days in a year and, longer inundating duration would lead to the replacement of flood-tolerant species by native shrub swamp species [48].

From the current data, the seed bank of typical bottomlands in the study area includes *Mazus japonicus*, *Saluia plebeian*, *Phalaris arun dinacea*, *Phragmites australis*, and *Cyperus difformis*. All of these can be divided into submerged aquatic, emergent, and amphibious vegetation. According to the researches of Chen et al., *Phragmites australis* is dominant on the floodplains all along the Yangtze River [49] and is capable of developing new plants all over the year. The simulation results of the Kootenai River by R.Benjankar et al. show that once the original environment gets interfered by dam construction or grazing, *Phragmites australis* and grass usually become the major species [50]. However, the slight decrease of plants in Jiaoziyuan in 2011 indicates that when an arid year led to a sharp decrease in submerging time (from 79 to 19 days), original dominant communities would be disturbed and thus resulted in the area decrease. After the dam construction, the two gauge stations downstream of the Sabine River represent different changes in the runoff, and the lower floodplains, which get longer flooding duration showed similar vegetation community composition [51]. Thus, dam operations could indirectly trigger certain seeds to burgeon to some extent. This is consistent with the trends seen in the Elwha River and Sauce Grande River under the influence of large dams [52]. However, research concerning the Yangtze River has been chiefly focused on Dongting Lake and Poyang Lake rather than on the floodplains.

Apart from natural occupancy, some bottomlands have been reclaimed for agriculture, including Liutiaozhou, Mayangzhou, Huojianzhou, and the higher portions of the rest of those studied. Farmers use geometric shapes to manage crops, which leave visible ribbing on satellite images as evidence of human activities. The color denoting submergence times is normally red, meaning that these fields are flooded for fewer than 30 days. Therefore, vegetation coverage is surely in-line with crop types and the degree of human activities. If it is a goal of decision-makers to reduce vegetation cover to improve the capacity for the flood [53], then it is essential to understand what the most effective indicators are and adapt the methods based on different types of vegetation and occupancy patterns. For floodplains of high elevations, guiding the flooding process upon them is far from practical. Thus, in order to manage the activities of farmers that can impact the watershed, compulsory policies are necessary. As for low-elevation floodplains, current runoff has prevented the plants from rooting and growing but still needs observation on the submergence time as the flood volume changes. From Fig 11, we found that plants have grown since the inundation duration decreased into proper range (<120 days) and been developed abundantly under the suitable circumstance in Guanzhou, Jiaoziyuan, and Tuqizhou. Those places still remain within the water fluctuation range, which makes it possible to either promote the sediment erosion to lower the elevation or raise the flood volume to lengthen the submerging time [48]. Throughout all the floodplains that have the characteristics of Group Ⅲ like Jiaoziyuan, many have formed a unique land view [49], and dominant vegetation has emerged due to the consistent and steady hydrodynamic conditions. By field surveys, it is realistic to summarize the adaptations for the dominant species. Therefore, more detailed indicators could be explored and established in such places from more effective perspectives [54].

Managing the floodplain vegetation is a complex project, including muti-objectives like river adjustment and vegetation growth characteristics. Our study provides insights into the bond-to-submergence time and vegetation coverage from a hydrodynamical viewpoint while other factors were not taken into consideration, such as temperature and humidity. To understand how large-scale vegetation influences large floods, vegetal types must be identified as submerged aquatic, emergent, or amphibious vegetation. Once the respective mechanisms are confirmed, the roughness caused by these plants may be accounted for in numerical models to improve flood predictions and reservoir management.

## Supporting information

S1 Fig Time series of annual water level at Zhicheng, Shashi, Xinchang, Shishou and Jianli stations. (TIF)

S1 File The average elevation of the 11 floodplains from 1998–2015. (CSV)

S1 Graphical abstract (TIF)
