# Supplementary figures and images for: Response of vegetation to submergence along Jingjiang Reach of the Yangtze River
Source: PLoS One. 2021 May 7;16(5):e0251015. doi: 10.1371/journal.pone.0251015 (PMC8104387; doi:10.1371/journal.pone.0251015)

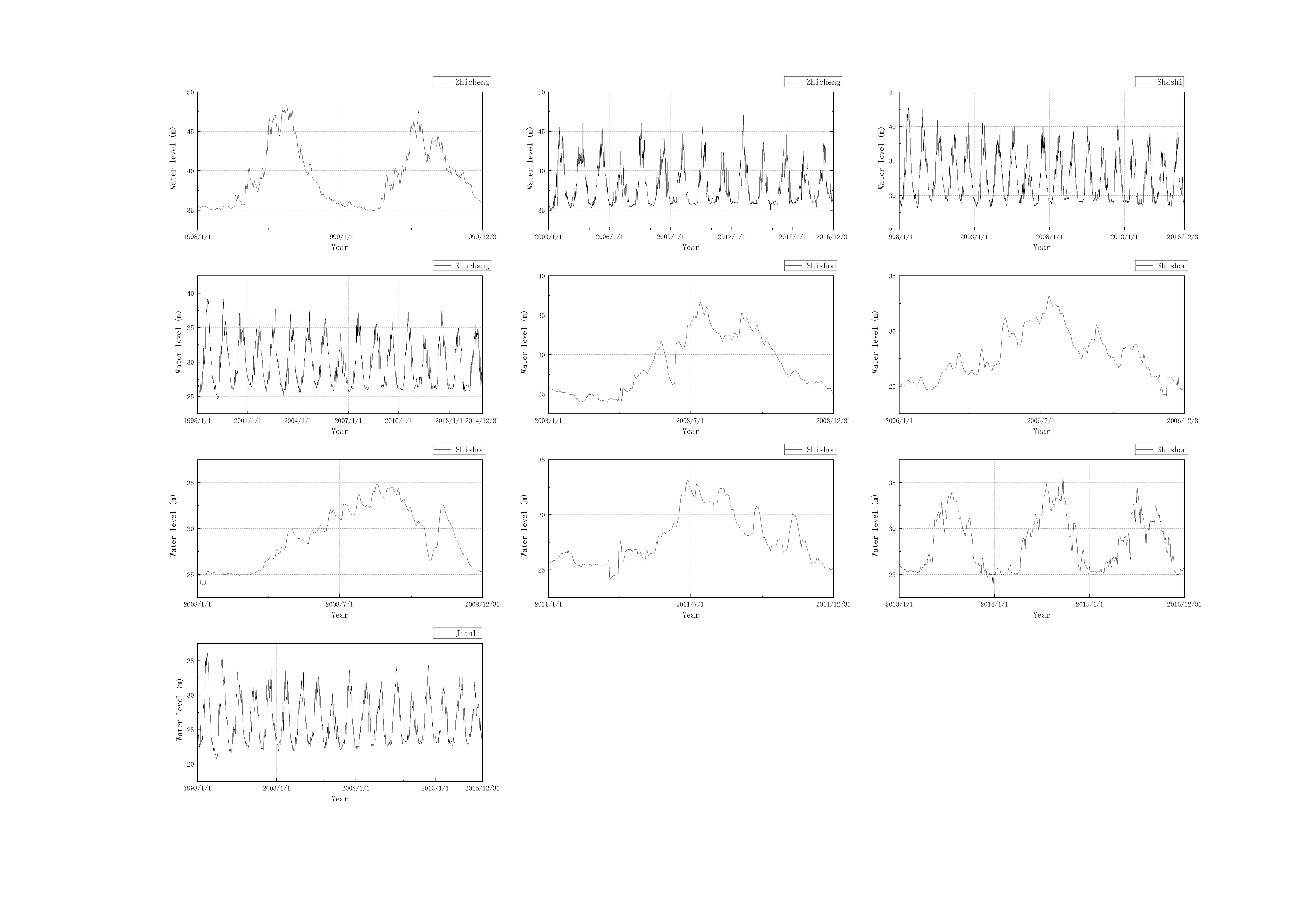

Supplement: S1 Fig — (TIF) [file pone.0251015.s001.tif]

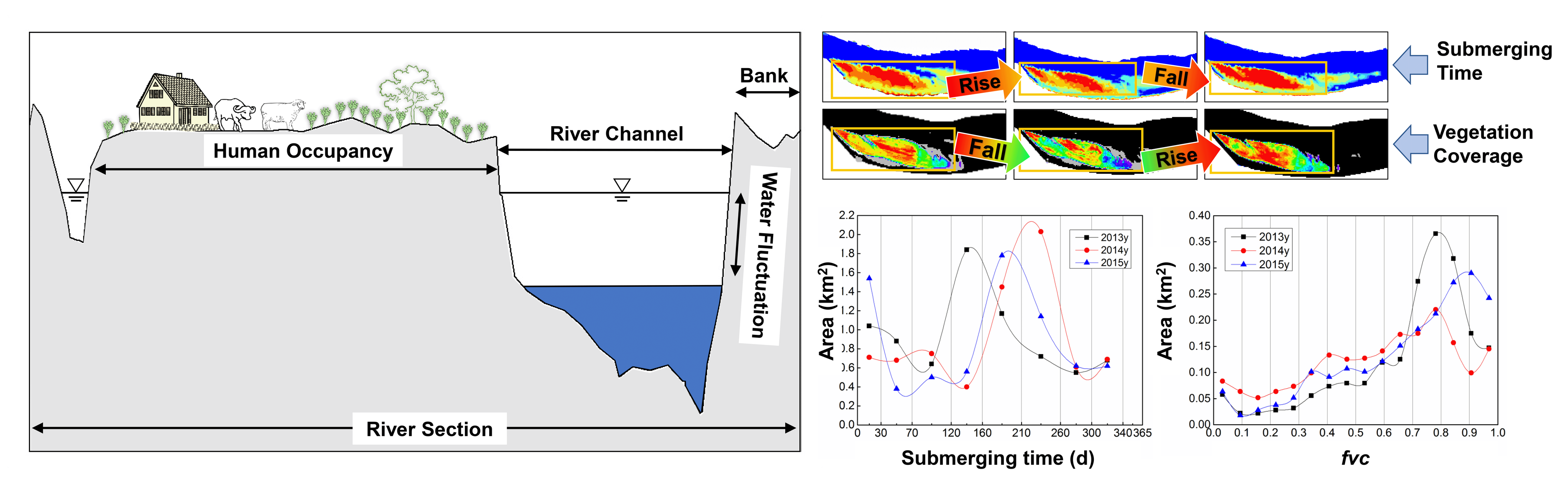

Supplement: S1 Graphical abstract — (TIF) [file pone.0251015.s003.tif]
